# GABA and the GABA_A_ Receptor

**Published:** 1997

**Authors:** S. John Mihic, R. Adron Harris

**Affiliations:** S. John Mihic, Ph.D., is an assistant professor in the Department of Physiology and Pharmacology, Bowman Grey School of Medicine, Winston-Salem, North Carolina. R. Adron Harris, Ph.D., is a professor in the Department of Pharmacology and director of the Alcohol Research Center, University of Colorado Health Sciences Center, and a research career scientist at the Denver Veterans Administration Medical Center, Denver, Colorado

**Keywords:** GABA, GABA receptors, neurotransmission, brain, sedative hypnotics, receptor proteins, chloride channel, ion, protein kinases, AOD dependence, AOD tolerance, AOD intoxication, AOD use susceptibility, animal model, literature review

## Abstract

The neurotransmitter gamma-aminobutyric acid (GABA) inhibits the activity of signal-receiving neurons by interacting with the GABA_A_ receptor on these cells. The GABA_A_ receptor is a channel-forming protein that allows the passage of chloride ions into the cells. Excessive GABA_A_ activation may play a role in mediating the sedative effects of alcohol and other sedating and anesthetic agents. For example, alcohol enhances the GABA_A_-mediated chloride flow into cells and may thereby enhance neuronal inhibition. Alcohol’s effects on the GABA_A_-receptor function likely involve other molecules (e.g., other neurotransmitters and proteins that add phosphate groups to the receptor [i.e., protein kinases]). Several experimental approaches also have suggested that changes in GABA_A_-receptor function contribute to the tolerance to and dependence on alcohol. Finally, individual differences in the GABA system may play a role in determining a person’s susceptibility to developing alcohol dependence.

Nerve cells, or neurons, in the brain communicate through chemical messengers called neurotransmitters. These molecules are released by the signal-emitting neuron and bind to specific proteins (i.e., receptors) on the signal-receiving neuron. (For more information on signal transmission within and among nerve cells, see the article “The Principles of Nerve Cell Communication,” pp. 107–108.) Two main types of neurotransmitters and neurotransmitter receptors—excitatory and inhibitory—determine the response of the signal-receiving neuron. Excitatory neurotransmitters and their receptors increase the neuron’s intrinsic electrical activity and excitability, whereas inhibitory neurotransmitters and their receptors reduce neuronal excitability. For optimal functioning, the brain must balance the excitatory and inhibitory influences: Excessive excitation can lead to seizures, whereas excessive neuronal inhibition can result in incoordination, sedation, and anesthesia.

Gamma-aminobutyric acid (GABA) is the primary inhibitory neurotransmitter in the central nervous system. Because alcohol intoxication is accompanied by the incoordination and sedation indicative of neuronal inhibition, researchers have investigated alcohol’s effects on GABA and its receptors. This article summarizes findings that alcohol significantly alters GABA-mediated neurotransmission and presents some evidence that the primary GABA receptor (called the GABA_A_ receptor) may play a crucial role in the development of tolerance to and dependence on alcohol as well as contribute to the predisposition to alcoholism.

## The GABA_A_ Receptor

GABA_A_ receptors are large proteins^1^ embedded in the cell membranes of neurons (see figure). Each receptor consists of five protein molecules, or subunits, that assemble so that a channel is formed at the center of the complex. When GABA molecules or GABA-like compounds bind to the receptor and activate it, this channel temporarily opens and allows the passage of negatively charged molecules (i.e., ions), such as chloride ions (Cl^−^), to pass from the cell’s exterior to its interior. This ion flow decreases the cell’s excitability. The cumulative neuronal inhibition caused by GABA’s binding to many neurons results in sedation and intoxication (Whiting et al. 1995). In laboratory animals, these effects manifest themselves as loss of the righting reflex—that is, the animals can not get up when placed on their backs. Compounds that enhance the GABA_A_ receptor’s activity cause increased neuronal inhibition. In contrast, compounds that reduce GABA_A_ receptor activity result in the excitation of the signal-receiving neurons.

The subunits that constitute the GABA_A_ receptor each consist of a large extracellular region located on the outside of the cell membrane, four segments spanning the cell membrane, and several intracellular regions that are exposed to the neuron’s interior. Whereas the extracellular protein region is responsible for GABA binding, the intra-cellular regions can be modified by the addition of phosphate groups (i.e., can become phosphorylated). As described later in this article, this phosphorylation, which is performed by enzymes such as protein kinase C (PKC) and occurs at specific sites of the GABA receptor subunits, regulates the receptor’s functioning.

Many different GABA_A_ receptor subunits have been identified. These fall into three groups: α, β, and γ subunits. Each of these groups contains several different subunits (e.g., γ_1_ and γ_2_). The exact subunit composition of most GABA_A_ receptors is not known. Most likely, each receptor consists of two α subunits, one β subunit, and two γ subunits (see figure). Each subunit type only interacts with specific molecules. Thus, the α and β subunits can interact with GABA, whereas the α and γ subunits contain the binding site for benzodiazepines (see below). Different subunits within each of the three groups also differ in their pharmacological properties (e.g., the sensitivity to alcohol). Consequently, the specific subunit composition of each GABA_A_ receptor molecule determines that receptor’s overall characteristics. GABA_A_ receptors in different neurons or brain regions or at various developmental stages therefore can differ in their pharmacological properties (McKernan and Whiting 1996).

GABA_A_ receptors are found throughout the brain. This wide distribution likely is responsible for the plethora of behaviors (e.g., sedation, relief of anxiety, and motor in-coordination) produced by agents that activate these receptors, such as alcohol.

## The GABA_A_ Receptor’s Role in Alcohol Intoxication

Numerous clinically useful sedating medications (e.g., benzodiazepines, such as Valium^®^, and barbiturates, such as phenobarbital) and anesthetic agents (e.g., halothane) exert their effects at least in part by enhancing GABA’s influence on GABA_A_ receptors. Thus, these agents tilt the balance of excitatory and inhibitory influences in the brain toward inhibition, thereby causing the incoordination, sedation, and even anesthesia that accompany their use. Because alcohol produces similar effects, it also likely promotes neuronal inhibition through the GABA_A_ receptor (Tabakoff and Hoffman 1996).

Using several different approaches, researchers have attempted to determine which of alcohol’s behavioral effects are mediated by changes in GABA_A_ receptor function. One strategy has been to administer alcohol together with other compounds that interact with the GABA_A_ receptor and then determine whether alcohol enhances or impedes the effects of these compounds. For example, injections of GABA or GABA-like compounds into the brains of rats increased alcohol’s incoordinating and hypnotic effects (Deitrich et al. 1989). Similarly, rats that were treated with a compound that inhibits GABA degradation exhibited increased alcohol-induced incoordination (Deitrich et al. 1989). Finally, a compound called Ro 15-4513, which inhibits GABA_A_ receptor function, has been shown to prevent some of alcohol’s behavioral effects. For example, Ro 15-4513 reduced the severity of alcohol’s hypnotic effects and decreased alcohol consumption in animals (Mihic and Harris 1996). Such studies, however, provide only indirect evidence of alcohol’s actions and therefore must be interpreted with caution.

More direct evidence of alcohol’s interaction with the GABA_A_ receptor derives from neurochemical analyses and from studies in mouse and rat strains bred to differ in their sensitivities to some of alcohol’s behavioral effects. Neurochemical studies have analyzed alcohol’s effects on GABA-mediated Cl^−^ uptake into brain “microsacs”—membranes isolated from brain cells that form sealed bags—and spinal-cord neurons grown in tissue culture. Many of these studies found that alcohol increased Cl^−^ uptake, suggesting that alcohol could enhance GABA-mediated inhibition of neurons (Mihic and Harris 1996).

Researchers also have investigated alcohol’s effects on GABA_A_ receptor function in mouse and rat strains specifically bred to differ in their susceptibilities to alcohol-induced incoordination or loss of righting reflex. For example, so-called long-sleep (LS) mice exhibit a longer duration of the loss of righting reflex after an acute alcohol injection than do short-sleep (SS) mice. Studies in these mice found that alcohol enhanced GABA-mediated Cl^−^ uptake into brain microsacs obtained from LS mice but not into microsacs obtained from SS mice (Mihic and Harris 1996). These findings suggest that a biochemical difference in alcohol’s effects on the GABA_A_ receptor may underlie the behavioral differences observed between the two strains.

Alcohol’s effects on GABA_A_ receptor function likely involve the actions of other cellular proteins, such as the PKC enzymes that phosphorylate the GABA_A_ receptor at specific sites. In one experiment, for example, mice lacking a certain PKC subtype in the brain displayed reduced sensitivity to alcohol on several behavioral tests. Moreover, alcohol no longer enhanced the GABA-induced flow of Cl^−^ into brain microsacs prepared from these PKC “knock-out” mice (Mihic and Harris 1996). This observation further strengthens the hypothesis that alcohol-induced enhancement of GABA_A_ receptor activity not only involves proteins other than the receptor proteins but also requires protein phosphorylation.

Other studies have used electrophysiological techniques to assess alcohol’s effects on GABA_A_ receptor function. These studies have employed different experimental systems: (1) neurons that are still in an intact brain, (2) neurons in thin slices of isolated brain tissue, (3) isolated brain cells that have been grown in tissue culture, and (4) nonneuronal cells that normally do not produce GABA_A_ receptors but which can be induced artificially to manufacture receptors composed of specific subunits. Like the experiments described previously, these electrophysiological analyses indicate that the mechanisms underlying alcohol-induced enhancement of GABA-mediated signal transmission are complex and may involve neurotransmitter receptors other than the GABA_A_ receptor. For example, one study found that alcohol enhanced the activity of the GABA_A_ receptor on certain cells in the cerebellum of rats only in the presence of the neurotransmitter norepinephrine, which acts through another receptor, the β-adrenergic receptor (Freund and Palmer 1997). These findings suggest that alcohol-dependent enhancement of GABA activity in the cerebellum requires the activation of the β-adrenergic receptor. This receptor is located on the same cells in the cerebellum as the GABA_A_ receptor. Both receptors also interact in the absence of alcohol, but this interaction may be enhanced in the presence of alcohol.

At least three plausible mechanisms could explain the interactions among the β-adrenergic receptor, the GABA_A_ receptor, and alcohol, as follows:

- Norepinephrine could increase the GABA_A_ receptor’s sensitivity to alcohol.
- Alcohol could interact with the β-adrenergic receptor, thereby increasing that receptor’s ability to modulate GABA_A_ receptor function.
- Alcohol may further increase β-adrenergic enhancement of GABA_A_ receptor function by inhibiting the removal of norepinephrine from the synapses.

β-adrenergic signal transmission results in increased protein phosphorylation. Thus, whatever the exact mechanism may be, the association between the activities of the GABA_A_ and β-adrenergic receptors supports the conclusions from the C1^−^-flow analyses described above that alcohol’s effect on the GABA_A_ receptor may require activation of phosphorylating proteins, such as PKC (for a detailed discussion, see Weiner et al. 1997).

The link between protein phosphorylation and the sensitivity to alcohol of the GABA_A_ receptor also has been confirmed in studies analyzing alcohol’s effects on GABA_A_ receptors with known subunit composition. For example, the γ_2_ subunit of the GABA_A_ receptor exists in two forms—a short variant (γ_2S_) and a long variant (γ_2L_)—which differ in size by eight amino acids. Analyses in cultured cells found that receptors containing the γ_2L_ subunit showed alcohol-induced enhancement of their activity, whereas receptors containing the γ_2S_ subunit generally were insensitive to intoxicating alcohol concentrations (Wafford and Whiting 1992; Whitten et al. 1996; Harris et al. 1997). The additional eight amino acids present in γ_2L_ contain a site that can be phosphorylated by PKC, indicating that phosphorylation is a prerequisite for the GABA_A_ receptor’s sensitivity to alcohol. However, these experiments only can be performed in cultured cells or other artificial systems, not in intact brains. Therefore, one cannot conclude un-equivocally from these studies whether the GABA_A_ receptor’s sensitivity to alcohol in an intact organism is determined by differences in receptor subunits, phosphorylating enzymes, or other unknown factors (see Weiner et al. 1997; Harris et al. 1997; Mihic and Harris 1996).

**Figure:**
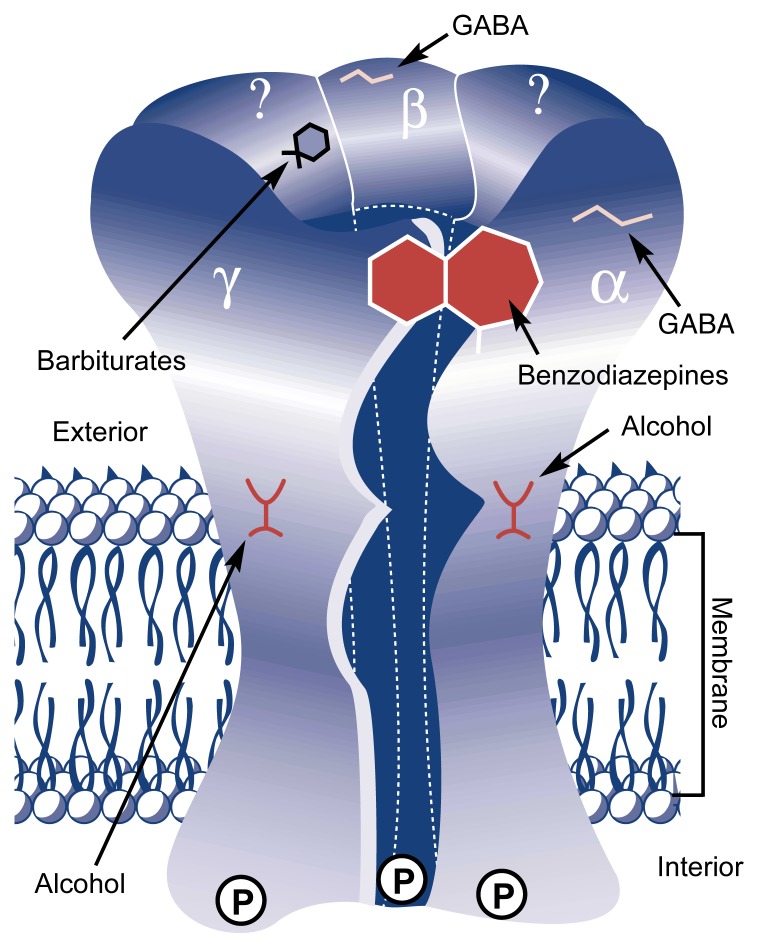
Schematic representation of the gamma-aminobutyric acid (GABA_A_) receptor. The functional receptor consists of five proteins, or subunits—most likely two α subunits, one β subunit, and two γ subunits. (Question marks indicate that the identity of these subunits has not been confirmed.) The proposed binding sites for GABA (α and β subunits), benzodiazepines (adjacent α and γ subunits), barbiturates (unidentified subunit), and alcohol (α, β, and γ subunits) are indicated. P’s represent phosphate groups attached to the receptor that regulate the receptor’s activity and sensitivity to alcohol.

## The GABA_A_ Receptor and Alcohol Tolerance and Dependence

After continuous alcohol consumption, both humans and laboratory animals develop tolerance to alcohol’s effects—that is, they require larger amounts of alcohol to achieve the same effects. Moreover, continuous alcohol consumption leads to the development of dependence, which is manifested by certain behavioral and physiological withdrawal responses that occur when alcohol is withheld (e.g., anxiety, excitability, and seizures). Changes in GABA_A_ receptor function may help explain both tolerance and dependence. For example, changes in the GABA_A_ receptor that would reduce its susceptibility to alcohol’s effects could produce tolerance. Similarly, inhibition of GABA_A_ receptor function may occur during alcohol withdrawal, because medications that inhibit the receptors’ activity (i.e., receptor antagonists) produce symptoms similar to those observed in alcoholics during withdrawal. Several experimental approaches have been used to investigate the role of the GABA_A_ receptor in alcohol tolerance and dependence. These approaches include studies of receptor antagonists, biochemical and electrophysiological analyses, and genetic analyses.

The effects of GABA_A_ receptor antagonists were studied in animals that underwent a regimen of chronic alcohol administration followed by alcohol withdrawal to induce seizures in the animals. The alcohol-treated animals were more susceptible to the seizure-inducing effects of a GABA_A_ receptor antagonist called bicuculline and of a compound called picrotoxin, which inhibits chloride channels (including the GABA_A_ receptor), than were animals that had not received alcohol (Buck and Harris 1991; Morrow 1995). These findings indicate that chronic alcohol administration had reduced GABA_A_ receptor function, so that lower levels of the GABA_A_ receptor antagonists were required to induce seizures.

The effects of chronic alcohol treatment on GABA_A_ receptor function also have been examined biochemically and electrophysiologically. Several studies found that whereas one-time alcohol administration enhanced GABA-induced Cl^−^ flow into mouse brain microsacs, no such effect occurred after chronic alcohol administration (Buck and Harris 1991; Morrow 1995). This resistance to alcohol’s chronic effects may represent a mechanism for alcohol tolerance. Similarly, the ability of benzodiazepines to enhance GABA-induced Cl^−^ uptake into brain microsacs was reduced in microsacs obtained from mice that had received chronic alcohol treatment, suggesting that chronic alcohol administration induced tolerance not only to alcohol but also to other substances affecting the GABA_A_ receptor (Buck and Harris 1991).

One potential mechanism underlying alcohol tolerance at the cellular level is a decrease in the number of GABA_A_ receptors on each neuron to compensate for the continuous alcohol-induced increase in GABA_A_ receptor activity. To investigate this hypothesis, researchers have monitored changes in GABA_A_ receptor subunit levels after chronic alcohol administration by determining the messenger ribonucleic acid (mRNA) levels for these subunits. mRNA is an intermediate molecule produced during the conversion of the genetic information encoded in the DNA into a protein product (e.g., a GABA_A_ receptor subunit). Whereas the DNA levels in each cell remain constant, mRNA levels fluctuate. Thus, mRNA levels increase when the gene is “turned on” and much protein is produced. Conversely, mRNA levels decrease when the gene is “turned off” and only little protein is produced.

Several recent studies have investigated the effects of chronic alcohol administration on the levels of mRNA for various GABA_A_ receptor subunits. For example, analyses in rats found that chronic alcohol treatment leads to reduced mRNA levels for one of the α subunits (i.e., the α_1_ subunit) as well as to decreased α_1_ protein levels (Morrow 1995). These findings support the hypothesis that tolerance development involves reduced GABA_A_ receptor numbers. The levels of other GABA_A_ receptor subunits, however, appear to be elevated. Furthermore, studies in humans produced conflicting results regarding the levels of α_1_ mRNA, possibly because analyses in humans often cannot be controlled as accurately as in animals. Additional studies found that the α_1_ mRNA levels were reduced most significantly in animals that were genetically predisposed to severe withdrawal symptoms. These findings suggest that when alcohol is withheld, the reduced GABA_A_ subunit levels prevent GABA-induced signal transmission, thereby contributing to withdrawal symptoms, such as seizures.

## GABA and Alcohol Abuse and Dependence

Recent research findings suggest that the GABA system also may play a role in determining a person’s susceptibility to developing alcohol abuse or alcohol dependence. For example, one study compared the effects of the benzodiazepine lorazepam on the brain’s use of glucose (i.e., glucose metabolism) in nonalcoholic subjects with a family history of alcoholism (FP subjects) and subjects without such a family history (FN subjects) (Volkow et al. 1995). By measuring the glucose metabolism in various brain regions, researchers can determine whether these regions are active at the time of measurement (i.e., whether signal transmission is occurring). The study analyzed lorazepam’s effects in the cerebellum, an area at the base of the brain that is responsible for motor coordination. The FP subjects exhibited lower cerebellar glucose metabolism than did the FN subjects. Moreover, lorazepam reduced the cerebellar glucose metabolism to a lesser extent in FP subjects than in FN subjects. These findings suggest that the activity of the GABA_A_ receptors in the cerebellum was disrupted in the FP subjects, making these people less vulnerable to the actions of agents such as lorazepam or alcohol and thereby possibly promoting increased alcohol consumption.

Other studies found that alcoholics had fewer GABA_A_ receptors in various brain regions than did nonalcoholic control subjects (Freund and Ballinger 1988*a*,*b*). It is unclear, however, whether these reduced receptor levels were a cause or a consequence of chronic alcohol consumption. Researchers also found that some abstinent alcoholics had lower GABA levels in the blood, a finding that likely reflects reduced GABA levels in the brain (Adinoff et al. 1995). However, these low GABA concentrations apparently were not associated with an increased genetic predisposition for alcohol dependence. Finally, sons of alcoholics, who are at an increased risk of becoming alcoholics themselves, were more likely than control subjects to report feelings of intoxication following benzodiazepine consumption (Cowley et al. 1992, 1996). Together, these results suggest that some alcoholics may exhibit abnormal GABA metabolism. Moreover, GABA_A_ receptor function—as measured by benzodiazepine’s effects on brain metabolism and behavior—is disrupted less prominently in people with a family history of alcoholism and therefore may be related to these people’s genetic liability for alcoholism.

## Conclusions

Over the past decade, researchers have learned much about alcohol’s effects on GABA_A_ receptors. Evidence exists that both acute and chronic alcohol exposure alter GABA_A_ receptor function. Furthermore, these receptors may play important roles in the development of tolerance to and dependence on alcohol and may underlie some of the genetic differences in the susceptibility to alcohol’s actions. Understanding the molecular basis for alcohol’s effects on these receptors provides a fascinating research challenge. Perhaps the most perplexing question currently facing investigators who study alcohol’s interactions with GABA_A_ receptors is, Which factors determine whether a particular GABA_A_ receptor will respond to acute alcohol exposure? By answering this question, researchers will be able to elucidate the mechanism of alcohol’s actions not only on the GABA_A_ receptor but also on other neurotransmitter receptors in the brain that help mediate alcohol’s effects. Another pivotal question regards the mechanisms by which chronic alcohol consumption alters GABA_A_ receptor function. Knowledge of these processes should lead to new strategies for identifying people at risk for alcoholism and for treating the disease.

## Footnotes

1 For a definition of this and other technical terms used in this article, see central glossary, pp. 177–179.
